# Acute Feasibility of Neuromuscular Electrical Stimulation in Severely Obese Patients with Obstructive Sleep Apnea Syndrome: A Pilot Study

**DOI:** 10.1155/2017/3704380

**Published:** 2017-01-17

**Authors:** Isabelle Vivodtzev, Nicola A. Maffiuletti, Anne-Laure Borel, Angélique Grangier, Bernard Wuyam, Renaud Tamisier, Jean-Louis Pépin

**Affiliations:** ^1^Université Grenoble Alpes, HP2 Laboratory, Inserm U1042, Grenoble University Hospital, CS 20217, 38045 Grenoble Cedex 9, France; ^2^Human Performance Lab, Schulthess Clinic, Zurich, Lengghalde 2, 8008 Zurich, Switzerland

## Abstract

*Objective*. Obesity and obstructive sleep apnea (OSA) are closely interconnected conditions both leading to high cardiovascular risk. Inactivity is frequent and physical activity programs remain difficult in these patients. We investigated the acute feasibility of two neuromuscular electrical stimulation (NMES) modalities in extremely inactive obese patients with OSA.* Design*. A randomized cross-over study, with two experimental sessions (one per condition: multipath NMES versus conventional NMES).* Setting*. Outpatient research hospital.* Subjects*. Twelve patients with obesity, already treated for OSA.* Interventions*. No intervention.* Measures*. Feasibility outcomes included NMES current intensity, knee extension force evoked by NMES, and self-reported discomfort.* Results*. We found higher current intensity, a trend to significantly higher evoked force and lower discomfort during multipath NMES versus conventional NMES, suggesting better tolerance to the former NMES modality. However, patients were rapidly limited in the potential of increasing current intensity of multipath NMES.* Conclusion*. Both NMES modalities were feasible and relatively well tolerated by obese patients with OSA, even if multipath NMES showed a better muscle response/discomfort ratio than conventional NMES. There is an urgent need for a proof-of-concept study and interventional randomized controlled trials comparing NMES therapy versus current care to justify its utilization in obese and apneic patients with low physical activity levels.

## 1. Introduction

Obesity is a growing public health problem leading to increased cardiovascular risk [1]. Obstructive sleep apnea syndrome (OSA), leading to intermittent hypoxia and sleep fragmentation, is highly prevalent in patients with obesity [2] and synergistically participates towards the occurrence of cardiometabolic comorbidities [3]. Furthermore, inactivity, another common risk factor for multimorbidity, is frequent in overweight or obese patients with OSA [4], putting these patients at high risk of cardiovascular diseases.

The inability to withstand physical effort is central to the symptoms of both obesity and OSA. Obesity is associated with reduced respiratory compliance [5], lower cardiac performance at exercise due to cardiac lipotoxicity [6], and frequent osteoarthritis [7]. On the other hand, OSA is associated with a modified hemodynamic response during exercise [8], daily somnolence, and impairment of muscle (oxidative) energy metabolism [9], independently of the physical activity level of the patients. Effective treatment of OSA by continuous positive airway pressure (CPAP) or noninvasive ventilation (NIV) may represent an important target for improving cardiometabolic risk. However, both fail to reduce metabolic or inflammatory markers in obese patients with OSA [10] or obesity hypoventilation syndrome [11] and have no significant effect on spontaneous physical activity [12]. Spontaneous physical activity in these patients is very low, particularly in terms of intensity level [13]. This emphasizes the need to offer a combination of multiple modalities of treatment to this specific population of obese patients with treated OSA. Lifestyle interventions and training programs using various modalities of exercise to reduce inactivity have been extensively implemented. For many patients, however, the implementation of physical activity programs remains difficult for several reasons including the level of disability (further complicated by orthopedic disorders) and psychosocial causes (the scrutiny of others, depression, and the lack of specific equipment dedicated to the morbidly obese). Finally, sleep apnea is known to attenuate the effects of a lifestyle intervention program in men with visceral obesity [14].

Transcutaneous neuromuscular electrical stimulation (NMES) was originally introduced as a treatment modality to prevent deconditioning associated with immobilization, particularly for orthopedic patients [15]. As it has minimal ventilatory requirements, NMES is currently emerging as a promising alternative to general physical reconditioning in patients with advanced respiratory diseases who are either unable or unwilling to attend formal rehabilitation programs or to undertake reasonable spontaneous physical activity. NMES programs have been shown to enhance skeletal muscle mass and strength (+20% to +50%), particularly for the quadriceps muscle [16]. This would thus allow patients to better carry their body weight and minimize breathlessness during daily activities, which can in turn improve exercise performance and quality of life [16]. From a metabolic point of view, we and others have previously reported changes at the muscle molecular level in patients with chronic obstructive pulmonary disease (COPD) [17, 18]. NMES therapy significantly activated the (IGF-1/AKt) insulin signaling pathway in the stimulated quadriceps muscle [17], and the increase in muscle mass was comparable to that observed with voluntary exercise training [19]. Furthermore, NMES training may improve blood markers of insulin sensitivity in diabetes mellitus and obesity [20, 21]. These studies collectively suggest a potential protective metabolic effect of NMES therapy in overweight or obese patients although the exact molecular mechanisms remain to be determined.

A novel NMES paradigm has recently been introduced—multipath NMES—which has been shown to maximize quadriceps muscle recruitment while minimizing the discomfort associated with the exogenous stimulation both in healthy subjects [22] and in overweight orthopedic patients [23]. Therefore, the objective of this pilot study was to investigate the acute feasibility of multipath NMES and conventional NMES of the quadriceps femoris muscle in obese patients already treated for OSA. To this aim, NMES current intensity, knee extension force evoked by NMES, and self-reported discomfort were systematically compared between the two NMES modalities, which were randomly administered on two separate experimental sessions (cross-over design). We hypothesized that the use of multiple dynamically changing current pathways and large stimulating electrodes in multipath NMES would evoke stronger contractions (better recruitment) with less discomfort compared to conventional NMES [22].

## 2. Methods

### 2.1. Participants

#### 2.1.1. Inclusion

Twelve consecutive patients of the sleep department medical consultation in Grenoble University Hospital (Grenoble, France) were included in the study. Inclusion criteria were (1) adults with OSA based on apnea hypopnea index > 30/night at the time of diagnosis [24]; (2) being already treated for OSA by CPAP or NIV by more than 1 month with a compliance > 4 h per night; (3) obesity (35 < BMI < 45 kg/m^2^); and (4) extremely low physical activity levels (<5000 steps per day or less than 10 min walking/day). This trial was part of the ongoing longer-term study referenced as clinical trial NCT01820598. Ethical approval was obtained from the local ethical committee (CPP Sud Est V, France) and all the participants gave written informed consent.

#### 2.1.2. Characteristics

Patients' characteristics are reported in Table 1. The majority of patients were male (58%), aged 57 ± 10 years; they had a body mass index (BMI) of 40 ± 5 kg/m^2^ and a fat-free mass index (FFMI) of 22 ± 5 kg/m^2^, as assessed by bioimpedance analysis (Bodystat®1500 MDD Body Composition Monitor). They were well compliant to their nocturnal OSA treatment (by CPAP or NIV). Patient comorbidities were type 2 diabetes mellitus (33%), dyslipidemia (33%), and hypertension (66%). All patients were unfamiliar with NMES.

### 2.2. Experimental Procedures

**Figure 1:**
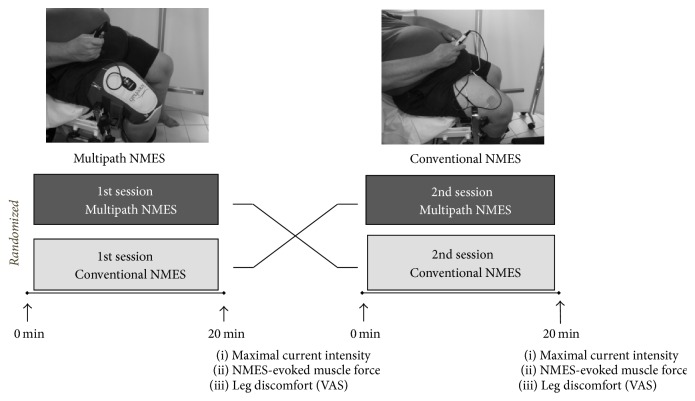
Overview of the experimental design: two 20 min sessions of NMES (one per condition: multipath NMES versus conventional NMES) were completed in a random order, with a cross-over design. In both conditions, stimulation frequency was 50 Hz, pulse duration was 400 *μ*s, and current intensity was self-determined at the maximal tolerable level. The two experimental sessions were separated by at least 48 h.

Twelve participants completed two identical experimental sessions, except for the type of NMES—multipath NMES (Kneehab®, BMR Ltd., Neurotech, Ireland) versus conventional NMES (Rehab 400 CefarCompex®, Scandinavia AB, Sweden)—that were randomly presented in a cross-over design (Figure 1). Experimenters and participants were both aware of which NMES modality was administered. Both NMES sessions lasted 20 min and were completed at the maximal tolerable intensity with the same stimulation frequency (50 Hz) and pulse duration (400 *μ*s). The experimental sessions were separated by at least 48 h and completed at the same time of day. Importantly, to mimic the real life condition at home, the patient increased by him-/herself the current intensity to his/her own maximal tolerable threshold throughout the experimental sessions.

#### 2.2.1. Multipath NMES

Multipath NMES was delivered with a two-channel Kneehab XP device (Bio-Medical Research, Galway, Ireland), which consists of a stimulation unit connected to a garment that wraps around the thigh and incorporates four large self-adhesive pregelled electrodes (10 × 20; 3 × 18; 10 × 7.5; and 7 × 14 cm).

#### 2.2.2. Conventional NMES

Conventional NMES was delivered with a portable and programmable stimulator unit (Compex 3, Compex Médical SA, Ecublens, Switzerland) connected to three self-adhesive pregelled electrodes (two active channels). Two 5 × 5 cm electrodes were positioned on the belly of the vastus lateralis and vastus medialis muscles. A large (5 × 10 cm) self-adhesive electrode was fixed on the gluteal crease to close the stimulation current loop.

### 2.3. Outcome Measures

The ability to increase NMES current intensity during a typical session (tolerance), the level of knee extension force evoked by NMES (as a percentage of the maximal voluntary contraction (MVC) force), and the level of discomfort associated with NMES were assessed in each condition.

#### 2.3.1. NMES Current Intensity

Current intensity was monitored by the patients themselves to ensure real life conditions. Participants were asked to progressively increase NMES current intensity as much as they could all along the session to attain a maximal sustainable intensity at midsession (10 min). This corresponded to a current level that they could sustain the remaining 10 min as well as the days after the session with the assumption that they would have continued NMES.

#### 2.3.2. Maximal Voluntary Contraction Force and Force Evoked by NMES

Isometric quadriceps MVC force was measured using a strain-gauge transducer (Sensy, 2712 model, Jumet, Belgium), a signal transducer, and specific software (ADInstruments PowerLab Systems), as previously described in our laboratory [13]. Patients were asked to sit on a custom-made bench with a 90° knee and hip flexions and to cross their arms over the chest; a strap was fixed over the waist to avoid strength overestimation. The strain gauge was posteriorly attached to the leg, 2-3 cm above the lateral malleolus. The highest force value of three reproducible contractions (<10% variability) was considered as MVC force. MVC force was measured 20 min before each NMES session (multipath NMES and conventional NMES) and the corresponding data are reported in Table 1. Evoked force was then measured on the same bench with the same gauge during series of contractions evoked by NMES, approximately 15 min after the beginning of each NMES session. The highest value of three reproducible measurements was considered as the evoked force by NMES.

#### 2.3.3. Discomfort

The level of discomfort associated with the application of NMES was assessed by a visual analogical scale (VAS) from 0 (no discomfort) to 10 (extreme/unsustainable discomfort), approximately 15 min after the beginning of each NMES session.

### 2.4. Analysis Methods

**Figure 2:**
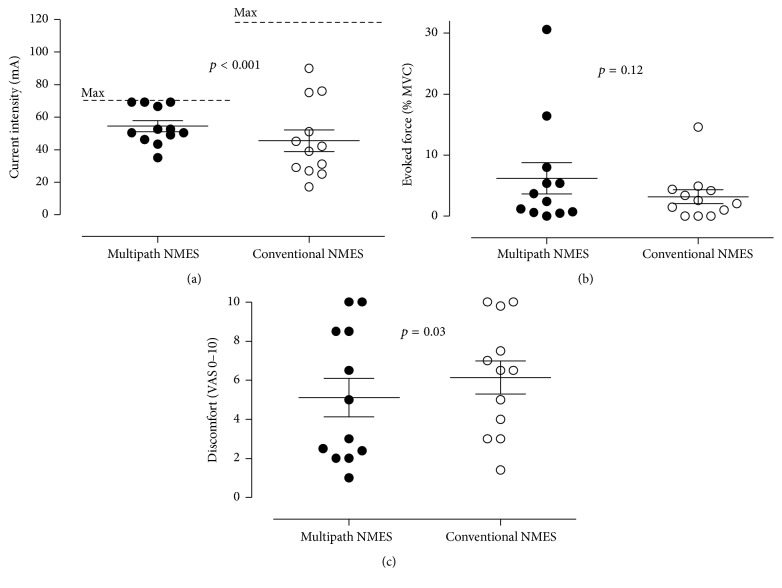
Acute responses to a single session of multipath versus conventional NMES in obese patients with OSA: comparison of individual values of (a) self-selected current intensity (dashed line: maximal current intensity delivered by each device); (b) NMES-evoked force; and (c) self-reported discomfort by stimulation modality (multipath versus conventional NMES, *n* = 12 for both treatment sessions). Bars are median and standard errors.

Continuous data were presented as mean and standard deviation (Table 1) or individuals' data with median and standard errors (Figure 2). For all tests, a significance level of 0.05 was used. Normality was checked using tests of Shapiro-Wilk and Skewness with a criteria of acceptance of −1.0 < Skewness score < 1.0. The comparisons of the variables (current intensity, evoked force, and discomfort) between the two conditions (multipath versus conventional NMES) were made by paired *t*-test or Wilcoxon signed ranks test for paired data depending on the normality of the data. All statistical analyses were performed using SAS 9.4 software (SAS Institute Inc., Cary, NC). These results have been presented at the American Thoracic Society meeting in 2016 [25].

## 3. Results 

### 3.1. Feasibility and Tolerance

All patients were able to complete the two NMES sessions with no adverse events. The highest current intensity reached during the session was 55 ± 12 and 46 ± 23 mA for multipath NMES and conventional NMES, respectively (*p* < 0.001; Figure 2(a)). The mean interval between m-NMES and c-NMES for current intensity was 8.9 ± 18.8 mA [min: −22.5, max: 30.3 mA].

### 3.2. Evoked Force by NMES

NMES-evoked force was 6.2 ± 9.0% MVC for multipath NMES versus 3.2 ± 4.0% MVC for conventional NMES (*p* = 0.12; Figure 2(b)). 42% (5 out of 12) and 17% (2/12) of the patients achieved a knee extension force greater than 5% and 15% MVC, respectively, with multipath NMES versus only 17% (2/12) and 8% (1/12) for conventional NMES. The mean interval between m-NMES and c-NMES for evoked force was 3.0 ± 8.5% MVC [min: −3.5; max: 30.6% MVC].

### 3.3. Discomfort

The level of discomfort was 5 ± 3 and 6 ± 3 for multipath and conventional NMES, respectively (*p* = 0.03; Figure 2(c)). This suggests a better tolerance to multipath NMES although patients were rapidly limited in the potential of increasing current intensity with this modality (see also Figure 2(a)), contrary to conventional NMES. The mean interval between m-NMES and c-NMES for the level of discomfort was −1.0 ± 1.7 [min: −4.5; max: 1.0].

### 3.4. Subjective Preference

Once completed the two experimental sessions, 8 patients declared having preferred the multipath NMES modality, 1 patient declared having preferred the conventional NMES modality, and 3 patients had no preference.

## 4. Discussion

The acute application of both multipath and conventional NMES was feasible with good tolerance in obese patients with OSA and visible evoked quadriceps muscle contractions in half of them. Importantly, acceptable knee extension force levels could be successfully achieved in some but not all patients, particularly with multipath NMES. This result is highly relevant because it proves that existing NMES technologies allow the electrical current to penetrate the subcutaneous adipose tissue and to attain the muscle, thus inducing visible contractions even in severely obese patients.

There are limitations to NMES implementation in obese patients directly related to their body composition. Excessive amounts of subcutaneous adipose tissue represent a physiological barrier that is likely to affect the potential effectiveness of NMES therapy for obese patients because of the increased distance between the stimulating electrode and the axon terminals and also because body fat is a poor conductor of electricity [26]. Therefore, high current intensities are required to induce visible muscle contractions in obese individuals, and this is quite likely to activate nociceptors in addition to muscle fibers, thus limiting the application of NMES in this specific population. Thus, as expected, current intensity levels found in the present study were much higher than previously reported in other chronic diseases such as COPD or heart disease [16, 27]. For a first session, the mean intensity level was about 45 mA as compared to 20 to 30 mA for similar NMES currents in COPD. The levels of force evoked by NMES were notably lower than those observed in healthy subjects at first NMES use [22], but actually close to those reported in other chronic diseases. In fact, with a mean of 6.2 ± 9.0% MVC for multipath NMES (but up to 16.4% and 30.6% of MVC in two patients), the force levels obtained in this pilot study and particularly with the multipath system were similar to those observed at first NMES application in patients with COPD [17, 28]. It is worth noting that an increase in current intensity is largely expected in the days following the very first NMES session, which could reach >+10 mA for the first week [28]. Our current results are thus of importance since we demonstrated that it was possible to evoke visible muscle contractions in about half of the patients when initially exposed to NMES. In patients with COPD, we previously reported that, after a 6-week NMES training, evoked force by NMES achieved a mean of 13 ± 10% MVC [17]. Even low, this amount of evoked force by NMES allowed an increase in muscle strength by 20% but also in muscle cross-sectional area by 6% in mean in these patients. Those results were linked to changes at the molecular level which suggested a successful stimulation of muscle protein synthesis [17]. Importantly both in our previous study in COPD and in the present study, patients monitored intensity level by themselves, suggesting that patients will be able to maintain those intensities at home.

It is not surprising however that not all the patients were able to reach high NMES current intensities during a single session [28]. In our study, almost half of the patients did not reach 6% MVC during their first NMES session. It is however likely that most of them will be able to double the current intensity in the first week of treatment [28]. The aptitude to tolerate high current intensities is extremely variable across patients, is individual specific [29], and depends on not only different uncontrollable factors such as skin and nerve sensitivity but also acceptance to discomfort [28]. Discomfort scores during the initial applications of NMES must be low to allow increasing current intensity during the whole treatment program, a condition for ensuring NMES effectiveness [17]. This aspect confirms the interest of multipath NMES for obese patients because of the lower discomfort with a trend for significantly higher evoked force, that is, a better muscle response/discomfort ratio as compared to conventional NMES. This is likely to explain why more patients subjectively preferred the multipath modality as compared to the conventional one (8 versus 1 out of 12).

### 4.1. Clinical Impact

Effective treatments of OSA for nocturnal respiratory troubles have been found to have little effect on cardiometabolic parameters in patients with OSA and likewise in obese patients who in addition cumulate metabolic comorbidities. In already CPAP treated patients, excluded for some reasons from usual exercise training program, combined strategies of care are necessary to counterbalance inactivity and muscle wasting. Until now, excessive amounts of subcutaneous adipose tissue have been considered as a physiological barrier affecting the potential effectiveness of NMES therapy in obese patients. With the recent clinical validation of the novel multipath NMES paradigm in an orthopedic population, which is able to maximize quadriceps muscle recruitment while minimizing the discomfort, we believe that NMES could be an interesting tool to increase the chance to maintain or even improve spontaneous physical activity in obese patients with OSA. Based on the result of the present study, we have initiated a larger, multicenter, and sham-controlled randomized trial to investigate the effect of m-NMES on spontaneous physical activity, functional parameters, and cardiometabolic parameters in severely obese patients with OSA.

Existing NMES devices show technical limitations (e.g., maximal current output, current distribution); thus they should be improved and adapted to the chronic disease population. Conventional NMES seems less appropriate for obese patients, due to limited muscle recruitment and high levels of discomfort. Multipath NMES is potentially appealing for this population also because of the excellent compliance for use at home (better than conventional NMES) [30], but the manufacturers should seriously consider the options to (1) increase the maximal current output from 70 mA to at least 100 mA to allow larger improvements in current intensity (and thus evoked force) within and between sessions and (2) extend the stimulation technology to muscle groups other than the quadriceps femoris (e.g., glutei, hamstrings) [21].

In conclusion, the acute application of NMES was feasible and well tolerated by obese patients with OSA and with a potential for molecular effects and muscle mass improvement (in case of repeated use), though it deserves to be proven in this population. There is an urgent need for a proof-of-concept study and interventional randomized controlled trials comparing NMES therapy versus current care to justify its utilization in obese and already treated apneic patients with low physical activity levels.

## Figures and Tables

**Table 1:** Patients' characteristics.

Variables	Mean	SD
Gender (M/F)	7/5	
Age (years)	57	10
BMI (kg/m^2^)	40	5
FFMI (kg/m^2^)	22	5
Waist circumference (cm)	113	15
Hip circumference (cm)	121	5
CPAP/NIV treatment	8/4	
CPAP/NIV compliance (h/night)	6	3
FEV_1_ (L/min)	2.3	0.8
MVC force		
Multipath NMES (kg)	43.0	28.4
Conventional NMES (kg)	44.5	27.7

BMI: body mass index; FFMI: fat-free mass index; CPAP: continuous positive airway pressure; NIV: noninvasive ventilation; FEV_1_: forced expiratory volume in one second; MVC: maximal voluntary contraction; NMES: neuromuscular electrical stimulation.
